# Surgery

**Published:** 1896-09

**Authors:** W. H. Harsant


### SURGERY.

It is only within the last few years that surgical relief has been thought of as applicable to **perforating ulcer of the stomach.** In 1883 Mr. Nelson C. Dobson lost a patient from gastric perforation. At the necropsy the opening was found to be in the anterior wall, and so accessible that he reported the facts,[1] and advocated in a similar case operative interference along one of the following lines: (1) Simple abdominal section with cleansing of the peritoneum, leaving the ulcer to heal of itself under rest and rectal feeding. (2) The closure of the perforation by suture, either with or without paring its edges. (3) The suture of the stomach, at the point of perforation, to the abdominal wall in order to establish a gastric fistula.

Dr. R. F. Weir and Dr. E. M. Foote, of New York, give an able summary[2] of **recent advances** in this branch of surgery. The first successful case reported was by Kriege,[3] and since that time the surgical treatment of perforating gastric ulcer has widely spread, particularly in Great Britain, so that now the collection of such operations has attained a total of seventy-seven cases, obtained from American, English, German, and French periodicals. Five-sixths of the seventy-eight cases occurred in women, three-fourths of whom were not above twenty-five years of age. With regard to the site of the perforation, it occurred forty-three times in the anterior wall, eleven times in the posterior wall, and six times in the lesser curvature. In twenty-seven cases the perforation was said to be near the cardiac orifice, and only nine times near the pylorus. The most important point in this connection is the accessibility of the perforation, and when we look over the tables with that idea in mind we find that in the large majority of cases it could be reached and sutured. In only eight cases is it stated that a suture of the perforation was impossible, either frcm its location, or the condition of the stomach-wall or that of the patient. This is by no means a bad record; on the contrary, it is an encouraging one. In sixteen cases the perforation was not found. In two of these it was considered unwise to separate adhesions between stomach and liver to look for it—a fatal caution. In several other cases the real cause of the peritonitis was not suspected, and in nearly every case, as the necropsies showed, the lesion was readily accessible to discovery and suture had a reasonably diligent search been made. In justice

[1] *Bristol M.-Chir. J.*, 1883, i. 196. [2] *Med. News*, 1896, lxviii. 449.
[3] *Berl. klin. Wchnschr.*, 1892, xxix. 1280.

be it said, however, that many of these cases occurred in the earlier days of this still new operation, and the operators had not the experience of others as their guide; also that the serious condition of the patient often limited the duration of a proper exploration.

An interesting point in connection with the surgical treatment of a gastric ulcer is the **time** which elapsed **between** the **perforation and** the **operation** done for its relief. It seems an invariable rule that perforation is attended with an attack of sharp abdominal pain, so that the time of perforation can usually be determined. The average time which elapsed before operation was thirty-six hours; but the extremes being so far apart as two hours and three hundred and thirty-six hours, the average has little value. It is more instructive to notice that in twenty-one cases which recovered the elapsed time averaged nineteen hours, while in two-thirds of these cases it was less than twelve hours. In the fifty-five unsuccessful cases the elapsed time averaged forty-three hours, and over twenty-four hours in two-thirds of these cases. It requires, therefore, no discussion to prove how much better is the chance of recovery when a perforation has existed ten hours than when it has existed twenty hours or more; and when a more certain diagnosis brings the patient sooner to the operating table, the percentage of recovery, which in the cases recorded up to date is about twenty-nine per cent., will doubtless be improved.

The secret of success lies in **early diagnosis.** There is almost invariably a history of gastric trouble, though not necessarily a well-marked history of gastric ulcer. There is an attack of sharp pain referred usually to the epigastric or left hypochondriac region, followed sometimes by pain in the lower abdomen. The pain is at first intense, often producing collapse or unconsciousness; usually it quickly moderates. Vomiting may or may not be present. Vomiting of blood is very unusual. In one case hæmatemesis had been present for many days; but although the vomiting continued after the perforation, it was without blood. After some hours, the time varying according to the amount and character of the escaped gastric contents, the symptoms of peritonitis manifest themselves. The physical examination of the patient is of great importance. In the earliest stage of the trouble the pallor, the anxious countenance, the feeble pulse, the thoracic respiration and the rigid abdomen, all point to the injury to the peritoneum. If gas has escaped from the stomach, or has developed by fermentation of the escaped gastric contents, the hepatic and splenic dulness may be reduced in area, or wholly disappear. It is evident that this condition might also follow intestinal perforation. But when the abdomen is opened the distinction between the two is plain, for the gas from the stomach is without odour, or slightly sour, while that from the intestines has a fæcal odour. As regards the operation itself, a median incision is the best, and should be of ample length, from three to five inches. The liver is to be lifted up by the hand of an assistant, and the stomach gently drawn downward and to one or the other side. Gentle squeezing of the organ will often expose the perforation by the escape of gas or fluid. If nothing is seen on the anterior surface, the lesser omentum is to be torn through at a thin place near the stomach, and the posterior wall explored as much as possible. With the aid of a sponge on a clamp through this same opening, the recesses of this cavity can be reached to search for extravasated fluid. If any recent adhesions exist about the perforation it is wiser to separate them, after protecting as far as possible the adjacent parts by gauze or sponges, and thus to find and to suture the gap, than to trust to the fibrinous closure. Excision of the ulcer has been advocated by some. On the other hand, it has been objected, with much reason, that this is unnecessary, that it takes valuable time, that it makes a larger and more difficult opening to close, and that hemorrhage follows, which is very detrimental to the patient and is often troublesome to check. In only three of the twenty-three successful cases was the ulcer excised. Of the remaining twenty, seventeen were sutured, Lembert style, while two of the others were tamponed, suture being impossible, and in the other case the fibrinous adhesions were not disturbed. In twelve of the successful cases irrigation of the peritoneal cavity was resorted to. In eight cases no irrigation was employed, but the peritoneum, when soiled, was cleansed with gauze. Five successful cases were drained; in ten no drainage was employed.

Dr. Alexis Thomson [1] records a **successful operation** for perforating ulcer of stomach some twenty-four hours after the first symptoms developed. The patient was a man aged 41, who had suffered from symptoms of gastric ulcer for five or six months. The perforation was attended with the usual attack of pain in the region of the umbilicus, vomiting and collapse. On opening the abdomen a circular hole was recognised on the anterior wall of the stomach, nearer the lesser than the greater curvature, and nearer the cardia than the pylorus. The hole was the size of a threepenny piece, and from it there escaped with each inspiration gushes of stomach fluid contents. While the fluid was soaked up with sponges a tube was passed down the œsophagus, and the remaining contents of the stomach were withdrawn. The walls of the stomach were then brought together with eight Lembert silk sutures. The peritoneal cavity was well washed out, and a drainage tube inserted, through which the fluid was sucked out every quarter of an hour for the first twenty-four hours. Four months after the operation the patient was strong and well. Dr. Alexis Thomson agrees with Mr. Pearce Gould that excision of the ulcer or paring of its edges is to be avoided. He recommends that a considerable area of stomach wall should be invaginated

[1] *Lancet*, 1896, ii. 11.

and that free washing out of the peritoneal cavity with hot saline solution or boiled water should be practised in all cases.

Dr. Charles T. Parker, of New York,[1] records a **successful case of this operation,** and points out that while medical treatment in these cases accomplishes practically nothing, laparotomy can alone assure one of the diagnosis and afford rational treatment. To be efficient it must be done early, and if the perforation is found and the peritoneum infected, one must make the necessary toilet of the peritoneum and suture directly, resect and suture, or perform a gastro-enterostomy. If the ulcer is difficult of access, and the above conditions cannot be carried out in their entirety, one is forced to suture and pack the cavity with gauze. The results of early laparotomy have been encouraging enough to force us to proceed in the attempt to make an early diagnosis, and to hope for even better results in the future than in the past. The results at the present day are certainly better than those of ten years ago. Pariser reports eleven recoveries in forty-three cases; Schuchardt reports two cases of recoveries; Heusner three cases which were fatal; Comte, who very lately collected the statistics upon this subject, has found nineteen recoveries in sixty-five cases. Dr. Parker's case was characterised by the symptoms of an antecedent gastritis, fixed pains in the epigastrium, and an observable induration in the epigastric region. The operation revealed an ulcer so near the point of perforation that the difference in tension within the abdomen brought about by the incision caused its rupture. The ulcer, which was on the anterior wall of the stomach, was so much indurated in its immediate surroundings, that it was thought better not to insert any sutures. The exposed area was accordingly washed out with a salt solution and packed with sterile gauze over which a sterile dressing was applied. The wound was dressed daily with the intention that as soon as the infection was over a suture of the stomach wall would be undertaken, if necessary. After the second day the discharge and odour from the cavity were diminished. This continued for one week, when the wound began to granulate. During the first five days following the operation the nourishment was entirely by the rectum. The temperature remained about 100.4°, and pulse 96. At the end of a week milk was given by the mouth. A fistula remained for some time, but healed in about three months, and complete recovery ensued.

Dr. R. A. Lundie, in January, 1895, showed a patient at the Edinburgh Medico-Chirurgical Society upon whom a **successful operation** had been performed for perforation of a gastric ulcer.[2] The patient was a dressmaker, aged 24, who had suffered from severe indigestion for about two years. The chief symptoms were pain, generally coming on an hour or less after taking food, and vomiting, which relieved the pain. No blood was

[1] *Ann. Surg.*, 1896, xxiii. 733. [2] *Edinb. Hosp. Rep.*, 1896, iv. 485.

ever vomited, so far as had been observed. For two months the symptoms had been severe and continuous. On October 17, 1894, while at work in the afternoon, she had a particularly sharp pain "at the bottom of the ribs" on the left side. It left her during the night, only to return the next day when she was on her way to work. In the afternoon and evening she remained at home and applied poultices, which she thought gave her some relief. On the 19th she went to sleep at night as usual, but was wakened about 1 a.m. by an agonising pain in the left side at the waist, which went across to the right side, and spread downwards all over the front of the abdomen. When seen at 9 a.m. she was lying on her back with knees drawn up, with a grey pinched face and an anxious expression, and complained of severe pain in the abdomen. Pulse 110, small and tense; temperature 99.6°. The abdominal walls were intensely hard and rigid all over, but especially in the upper part, yet there was less acute tenderness than might have been expected. Disappearance of liver dulness was not looked for. Operation was performed under chloroform at 11 a.m. the same day. A curved incision was made, with its extremities in the middle line, about 5½ in. apart, extending from a little below the xiphoid cartilage to a little below the umbilicus, with the convexity to the right, reaching at its centre to about 2½ in. from the middle line. The skin and subcutaneous tissues were thrown back, and the rest of the abdominal wall was divided in the middle line. As soon as the peritoneum was incised gas began to escape and yellow fluid to ooze out. The perforation was found to be a small circular opening, about ⅛ in. in diameter, on the anterior wall of the stomach. It must have been near the middle of the anterior surface, but the parts were not sufficiently exposed to allow this to be made out with precision. Though the coats around were indurated, there was no difficulty in doubling the peritoneal covering together. Fine silk was used; two stitches were passed through the perforation to prevent further leakage, and six Lembert sutures fixed the surrounding peritoneum. The stomach being quite flaccid, was not washed out. The abdomen was irrigated with a hot carbolic solution, about 1 to 120. The after progress of the patient was most satisfactory. She bore rectal feeding well, and had no nourishment by the mouth for more than a fortnight. When the wound was dressed for the first time, on October 29, it was found healed throughout by first intention, and all the stitches were removed.

W. H. Harsant.

